# Optimization of optical and mechanical properties of real architecture for 3-dimensional tissue equivalents: Towards treatment of limbal epithelial stem cell deficiency

**DOI:** 10.1016/j.actbio.2015.06.007

**Published:** 2015-09-15

**Authors:** Isobel Massie, Alvena K. Kureshi, Stefan Schrader, Alex J. Shortt, Julie T. Daniels

**Affiliations:** a Department of Ocular Biology and Therapeutics, UCL Institute of Ophthalmology, 11-43 Bath Street, London EC1V 9EL, UK; b Moorfields Eye Hospital NHS Foundation Trust, 162 City Road, London EC1V 2PD, UK; c Department of Ophthalmology, University of Düsseldorf, Moorenstrasse 5, 40225 Düsseldorf, Germany

**Keywords:** Cornea, Tissue equivalent, Limbal epithelial stem cell deficiency, RAFT, Amnion

## Abstract

Limbal epithelial stem cell (LESC) deficiency can cause blindness. Transplantation of cultured human limbal epithelial cells (hLE) on human amniotic membrane (HAM) can restore vision but clinical graft manufacture can be unreliable. We have developed a reliable and robust tissue equivalent (TE) alternative to HAM, Real Architecture for 3D Tissue (RAFT). Here, we aimed to optimize the optical and mechanical properties of RAFT TE for treatment of LESC deficiency in clinical application. The RAFT TE protocol is tunable; varying collagen concentration and volume produces differing RAFT TEs. These were compared with HAM samples taken from locations proximal and distal to the placental disc. Outcomes assessed were transparency, thickness, light transmission, tensile strength, ease of handling, degradation rates and suitability as substrate for hLE culture. Proximal HAM samples were thicker and stronger with poorer optical properties than distal HAM samples. RAFT TEs produced using higher amounts of collagen were thicker and stronger with poorer optical properties than those produced using lower amounts of collagen. The ‘optimal’ RAFT TE was thin, transparent but still handleable and was produced using 0.6 ml of 3 mg/ml collagen. Degradation rates of the ‘optimal’ RAFT TE and HAM were similar. hLE achieved confluency on ‘optimal’ RAFT TEs at comparable rates to HAM and cells expressed high levels of putative stem cell marker p63α. These findings support the use of RAFT TE for hLE transplantation towards treatment of LESC deficiency.

## Graphical abstract

**Figure:**
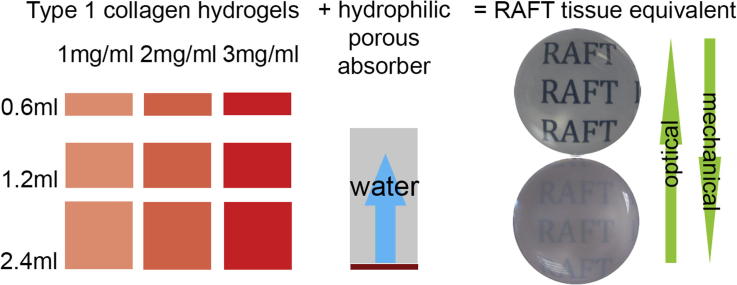

## 1 Introduction

The cornea is the uniquely transparent tissue located on the front of the eyeball that provides us with a window to the world. It is multilayered, consisting of an epithelium, Bowman’s layer, stroma, Descemet’s membrane and endothelium [1]. The stroma accounts for the majority of the thickness of the cornea but remains optically transparent due its highly organized structure, comprising optimally spaced, orthogonally arranged lamellae of collagen fibrils. This arrangement is tightly controlled to ensure that the stroma remains transparent throughout life, so that visual acuity is maintained, which is the major functional requirement of corneal tissue [2].

A continuously renewed epithelial cell layer protects the underlying stroma from external damage. Sloughed epithelial cells are repopulated by the progeny of limbal epithelial stem cells (LESC) that are located in the limbus, the vascularized border between central cornea and conjunctiva [3,4]. However, if LESC are damaged or lost, this can no longer occur. Inflammation, vascularization and ingrowth of neighbouring conjunctival cells follow and can lead to loss of corneal transparency [5].

One treatment for LESC deficiency is transplantation of pre expanded human limbal epithelial cells (hLE) on a carrier, such as human amniotic membrane (HAM) [6–8]. Although often effective, clinical graft manufacture using HAM can be inconsistent, perhaps due to its inherent biological variability [8–10]. Additionally, intra donor variation also exists whereby HAM samples isolated from different locations display different physical properties [11,12]. Further drawbacks of HAM are that before use, it must be screened, which is costly, and supply can also be unreliable [13].

As a result, many have aimed to develop materials that could be used for transplantation of hLE in place of HAM. Criteria for such a material should include capability to support hLE expansion along with appropriate optical and mechanical properties (i.e. the material should be as transparent as possible, but also be strong enough to withstand transplantation onto the recipients’ eye). Proposed materials range from naturally occurring materials such as fibrin, [14,15], fish scale collagen [16] and silk fibroin [17,18] to engineered polymers such as poly(lactide co glycolide) [19] and poly ε caprolactone [20].

Our approach is a tissue equivalent (TE), RAFT (Real Architecture for 3D Tissue), produced by gently wicking water away from Type 1 collagen hydrogels using hydrophilic porous absorbers [21]. Collagen is an attractive material for regenerative medicine applications as it is biocompatible, lowly immunogenic, can be remodelled by cells and is already used in numerous clinical applications (reviewed in [22]). We have previously shown that RAFT TEs can support hLE expansion and established RAFT TE as a good *in vitro* model of central cornea [23] and limbus [21]. The RAFT TE production process has also been through numerous iterations such that it is now reliable, robust and reproducible [21,23,24], which is desirable when developing tissue engineered products.

However, although promising in terms of biological function, we had not yet optimized RAFT TEs in terms of physical properties. Therefore, the aim of this study was to optimize the RAFT TE production process, which fortunately is tunable, to produce TEs with appropriate optical and mechanical properties for use in the treatment of LESC deficiency. RAFT TEs were compared to denuded HAM, which is a commonly used carrier for cultured hLE transplantation.

## 2 Materials and methods

### 2.1 Chemicals

All chemicals were obtained from Life Technologies, Paisley, UK, unless stated otherwise.

### 2.2 Preparation of RAFT constructs

Bovine dermis Type I collagen (Koken, Tokyo, Japan) (8 parts) was mixed with 1 part 10x Minimum Essential Medium (MEM) (Invitrogen). Collagen was used either neat (3 mg/ml) or prediluted using 1 mM hydrochloric acid (HCl) to 2 mg/ml or 1 mg/ml. Sodium hydroxide (5 M) was added dropwise to neutralize the solution to achieve a pH between 7.2 and 7.4. Finally, 1 part 1x MEM was mixed in carefully. This mixture was left on ice for 30 min to allow any air bubbles to disperse. The neutralized collagen mixture was pipetted into individual wells of a 24 well plate (Greiner, Stonehouse, UK) in volumes of either 2.4 ml, 1.2 ml or 0.6 ml, and heated to 37 °C for 30 min so that fibrillogenesis occurred, and a hydrogel formed. The majority of the liquid was wicked away from the hydrogels to produce RAFT TEs by application of hydrophilic porous absorbers (TAP Biosystems, Royston, UK) to the surface of the hydrogels for 30 min at 37 °C as described previously [21]. RAFT TEs were stored at 4 °C in phosphate buffered saline (PBS) before analysis. (For clarity, the protocol for RAFT TE production in previous studies was 2.4 ml of 2 mg/ml collagen [21,23]).

### 2.3 Preparation of human amniotic membrane samples

HAM samples with appropriate research consent were obtained from the University Eye Hospital (Heinrich Heine Universität, Düsseldorf, Germany). Ethical permission for this study was obtained from the Research Ethics Committee (UK) (reference No. 10/H0106/57-11ETR10). HAM samples from areas proximal and distal to the placental disc were isolated from 3 donors. In a laminar flow hood, intact HAM was washed with PBS to remove blood, before being stripped from the chorion. HAM was stored at −80 °C in 1x antibiotic, antimycotic/Dulbecco’s Modified Eagle Medium (100 IU/ml penicillin, 100 μg/ml streptomycin, 0.25 μg/ml fungizone). Prior to use, HAM was defrosted in a 37 °C water bath and washed for 3 × 10 min in Hank’s Balanced Salt Solution, once with agitation. HAM was oriented epithelial side up, and incubated with 0.25% trypsin ethylenediaminetetraacetic acid for 10 min and epithelium removed using cell scrapers. Denuded HAM was trephined using a 16 mm trephine (AngioTech, Vancouver, Canada) and stored in PBS in 24 well plates at 4 °C until analysis.

### 2.4 Subjective assessment of transparency

HAM and RAFT TE samples were placed over text (font: Cambria, size: 12), whilst still in a 24 well plate, still with 1 ml of PBS on top. Macroscopic photos were taken from a fixed distance using the same level of diffuse illumination.

### 2.5 Thickness measurements

The thickness of each RAFT TE and HAM sample was measured using optical coherence tomography (OCT). The PBS was aspirated and samples held in place between 2 glass coverslips. An OCT machine with anterior segment adaptor (HRA and OCT Spectralis, Heidelberg Engineering, Hemel Hempstead, UK) was used to image individual samples (10 line scans per sample). Images were opened using ImageJ software and scale calibrated. The line measurement tool was used to measure the thickness of the samples (all 10 scans per image were used for these measurements). OCT measurements were performed in triplicate for RAFT TEs and in duplicate for HAM samples within each experiment.

### 2.6 Transparency measurements

The PBS was aspirated from all samples and 14 masked observers assessed the transparency of each RAFT TE and HAM sample on 3 different days from a fixed distance using a standardized chart. Visual acuity of each masked observer was 20/20 or better with glasses or contact lens correction worn where required. An empty well of a 24 well plate was used as a control. The last line successfully read by each masked observer through control and test wells was recorded. When transparency of the sample was too poor to permit line 1 to be read, this was scored as 0 lines.

### 2.7 Light transmission measurements

Absorbance (400–700 nm) of RAFT TE and HAM samples with 1 ml of PBS on top was measured using a spectrophotometer (SAFIRE, Tecan, Reading, UK). A well containing 1 ml of PBS alone was used as a control. Absorbance readings were converted to percentage transmission using: % transmission = 10^–absorbance^ × 100 [25]. Absorbance readings were performed in duplicate for each RAFT TE or HAM sample within each experiment.

### 2.8 Mechanical property testing

#### 2.8.1 Quantitative

RAFT TE and HAM samples were removed from PBS storage and cut into “dog bone” shapes as described previously [26], 4 mm wide and 10 mm long, using a scalpel. Each end of the samples was clamped between metal mesh grips (MeshDirect, Burslem, UK) and loaded into a custom made tensile strength testing device, similar to that described previously [26]. Samples were held in place and weights applied incrementally until failure (breakage). The load at which failure occurred was recorded (any samples that slipped, instead of breaking, were excluded from analysis). Break stress was calculated using the following formula: break stress = force/cross sectional area [27]. (Cross sectional area was calculated using OCT thickness measurements.) This was performed in triplicate for each RAFT TE and HAM sample.

#### 2.8.2 Qualitative

Porcine eyes (FirstLink, Wolverhampton, UK) were transported to the laboratory on ice within 24 h post mortem. Excess skin, muscle and conjunctival tissue was removed. Eyes were disinfected by immersion into 2% povidone solution for 2 min, before a 1 min wash in 1x antibiotic, antimycotic in PBS. RAFT TEs (produced using 0.6 ml of 3 mg/ml collagen, 2.4 ml of 2 mg/ml collagen, and 2.4 ml of 3 mg/ml collagen) were transferred to the anterior surface of the eyes using forceps and glued in place using a fibrin glue, TISSEEL Lyo (Baxter, Norfolk, UK). Macroscopic photos were taken to demonstrate successful attachment and RAFT TEs were physically dragged to ensure attachment was secure.

### 2.9 Degradation study

HAM samples from 3 donors, and RAFT TEs produced using 0.6 ml of 3 mg/ml collagen were incubated in 1 ml of collagenase solution in DMEM (at either 10 mg/ml, 5 mg/ml or 1 mg/ml) at 37 °C for 24 h. As a control, samples were also incubated in DMEM alone (without collagenase). 100 μl aliquots of the solution were removed from each sample after 15, 30, 60, 240 and 1440 min. Photographs were taken at the same intervals. Collagen concentration in the solution was measured using the Total Collagen Assay (QuickZyme Biosciences, Leiden, Holland) according to the manufacturer’s instructions. All samples were diluted 1 in 10 in 4 M HCl before analysis, apart from those taken at 15 min, which were diluted 1 in 5, whilst blank and control samples were not diluted.

Results are expressed as percentage degradation compared to 24 h incubation in the same concentration of collagenase, calculated using the following formula:

$$(\text{collagen concentration at}\;t_{15\text{,}\;30\text{,}\;60\;\text{or}\;240}/\text{collagen concentration at}\;t_{1440})\times 100\text{.}$$

These experiments were performed in triplicate for each RAFT TE and HAM sample.

### 2.10 Preparation of human amniotic membrane for human limbal epithelial cell culture

Denuded HAM biopsies were cut into squares measuring 30 mm × 30 mm. The HAM was orientated so that the stroma was facing upwards. A sterile coverslip was placed on top and the free edges of the HAM biopsy folded around the coverslip. A second sterile coverslip was placed on top to secure the HAM in place. Mesh was removed from cell culture inserts (Millipore, Watford, UK) using a scalpel and the HAM biopsies placed on top. The top coverslip was then removed and edges of the HAM biopsy folded down around the cell culture insert. The final coverslip was carefully removed and the HAM secured in position using a suture. The cell culture insert was then orientated the correct way up (so that the stromal side of the HAM faced downwards).

### 2.11 Human limbal epithelial cell isolation and culture

6 cadaveric donor corneal rims with appropriate research consent were obtained from Moorfields Lions Eye Bank (UK). Ethical permission for this study was obtained from the Research Ethics Committee (UK) (Reference number 10/H0106/57-11ETR10). Corneas were stored at 31 °C in organ culture medium after enucleation and prior to hLE culture.

As previously [28], corneal rims were washed in 1x antibiotic/PBS (3 × 10 min). The superficial limbus was dissected away from the stroma and cut into 2 mm segments using fine sprung scissors and incubated overnight at 37 °C in a 5% CO_2_ in air incubator in 10 ml of 0.5 mg/ml collagenase type-L (Sigma–Aldrich, Dorset, UK). The next day, cells and tissues were pipetted up and down using a 10 ml pipette. The resulting cell suspension was centrifuged at 1000*g* for 5 min, and pellet washed using 3 ml of PBS. A second centrifugation was performed before the cell pellet was resuspended in culture medium (DMEM:MCDB-201 (3:2, Sigma–Aldrich), 2% foetal bovine serum, penicillin (100 IU/ml), streptomycin (100 μg/ml), gentamycin (50 μg/ml), AlbuMAX-I (1 mg/ml), l-ascorbic acid-2-phosphate sesquimagnesium salt (0.12 mM, Sigma–Aldrich), insulin transferrin selenium solution (1x), dexamethasone (10nM, Sigma–Aldrich), cholera toxin (100 ng/ml, Sigma–Aldrich), platelet-derived growth factor (10 ng/ml, R&D Systems, Abingdon, UK) and epidermal growth factor (10 ng/ml, Sigma–Aldrich)). The resulting cell suspension from each donor cadaveric rim was split either between 2 RAFT TEs (produced using 0.6 ml of 3 mg/ml collagen) and 2 HAM biopsies (*N* = 3), or split between 4 RAFT TEs (produced using 0.6 ml of 3 mg/ml collagen) (*N* = 3). Culture medium was changed 3 times per week and cells were maintained at 37 °C in a 5% CO_2_ in air incubator until confluency.

### 2.12 Wholemount immunohistochemistry

At confluency, RAFT TEs and HAM biopsies were fixed in 4% paraformaldehyde (PFA) (VWR, Leicestershire, UK) for 30 min, washed in PBS (3 × 5 min). Samples were blocked in 5% normal goat serum with 0.25% Triton X in PBS for 1 h. A capture antibody against putative stem cell marker p63α (Cell Signaling Technology, Herts, UK) was applied at 1:50 in 2% goat serum in PBS overnight at 4 °C. Isotype negative controls (Santa Cruz, Dallas, US) were included.

The next day, samples were washed in PBS (3 × 5 min). A secondary goat anti-rabbit 594 Alexa Fluor antibody was applied at 1:500 in PBS simultaneously with fluorescein isothiocyanate–phalloidin at 1:1000, to visualize cellular architecture, for 1 h at room temperature in the dark. Samples were washed in PBS (3 × 5 min) and mounted with 4′,6-diamidino-2-phenylindole (DAPI) mounting medium (Vector Labs, Peterborough, UK), onto glass slides. Confocal analysis was performed using a Zeiss LSM 710 microscope (Zeiss, Cambridge, UK).

### 2.13 Statistics

Results are expressed as average of 3 experimental repeats ± standard deviation, unless stated otherwise. GraphPad Prism software was used for statistical analyses. Significant differences between groups were tested using student’s *t*-test (2 groups) or one-way ANOVAs (more than 2 groups). For transmission, degradation, statistical analyses were performed after arcsine transformation [29]. Multiple linear regression analysis was used to determine correlation. For all statistical tests, *p* < 0.05 was considered statistically significant.

## 3 Results

### 3.1 Subjective assessment of transparency reveals that collagen concentration and volume affects RAFT TE appearance

**Fig. 1:**
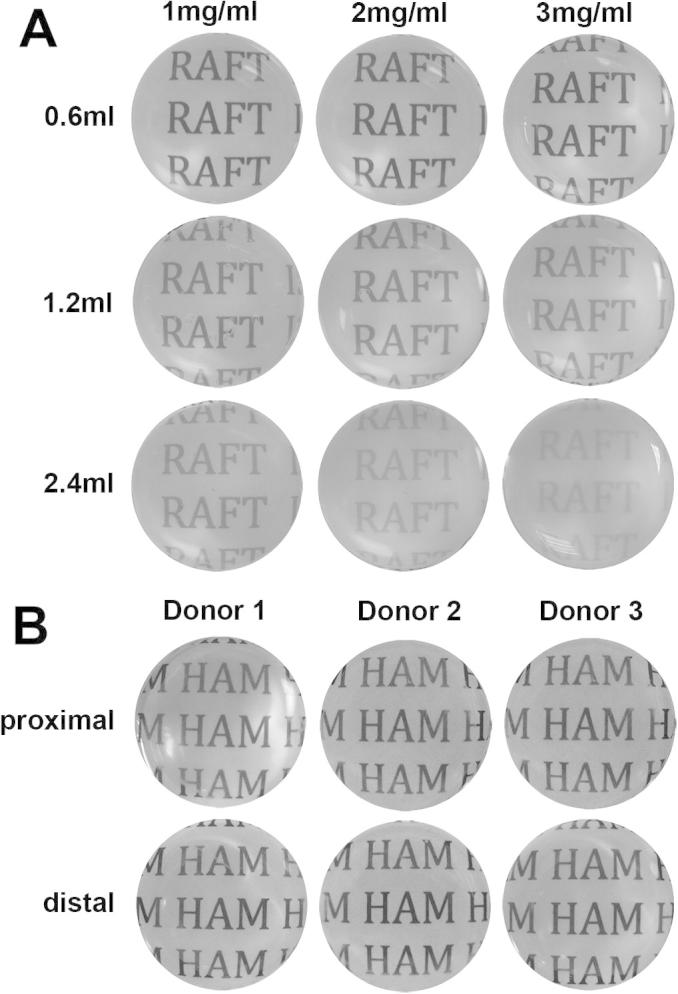
Subjective assessment of RAFT TE and HAM transparency. RAFT TEs were produced using different collagen volumes (0.6 ml, 1.2 ml or 2.4 ml) and concentrations (1 mg/ml, 2 mg/ml, or 3 mg/ml) and compared to proximal and distal HAM samples isolated from 3 donors. Macroscopic images of text through either RAFT TEs (A) or HAM (B) were captured for qualitative comparison.

Macroscopic photographs taken over text through either RAFT TEs or HAM samples revealed obvious differences in both RAFT TE and HAM optical properties (Fig. 1). Text could be read through all of the RAFT TEs but transparency appeared greatest for RAFT TEs made using 0.6 ml of 1 mg/ml collagen, whilst RAFT TEs made using 2.4 ml of 3 mg/ml collagen appeared least transparent. Subjective transparency was good for all 3 HAM donors and similar to RAFT TEs produced using 0.6 ml volumes of all 3 concentrations of collagen.

### 3.2 RAFT TE thickness is affected by both collagen volume and concentration, and is similar to that of HAM

**Fig. 2:**
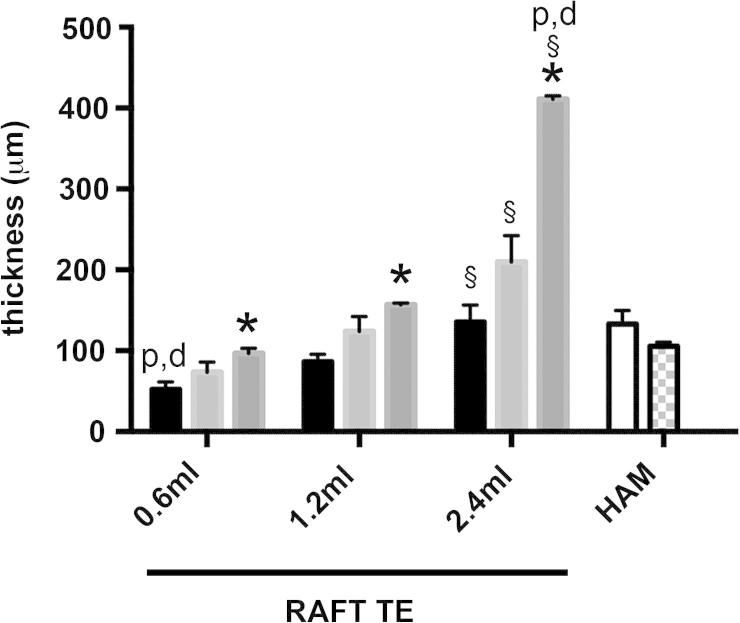
Thickness of RAFT TEs and HAM samples. RAFT TE and HAM sample thickness was measured using OCT. RAFT TEs were produced using either 0.6 ml, 1.2 ml or 2.4 ml of 1 mg/ml collagen (black bars), 2 mg/ml collagen (light grey bars) or 3 mg/ml collagen (mid grey bars) and compared with proximal (white bar) and distal (checkerboard bar) HAM samples. Data are averages from 3 experimental repeats ±SD. ^∗^*p* < 0.05 compared to RAFT TEs produced using the same volume of collagen but 1 mg/ml collagen. §*p* < 0.05 compared to RAFT TEs produced using the same concentration of collagen but 0.6 ml volume. d *p* < 0.05 compared to distal HAM samples. p *p* < 0.05 compared to proximal HAM samples.

RAFT TE thickness varied when different concentrations and volumes of collagen were used (Fig. 2). The thinnest RAFT TEs were produced using 0.6 ml of 1 mg/ml collagen (52.5 ± 8.9 μm), whilst the thickest RAFT TEs were produced using 2.4 ml of 3 mg/ml collagen (410.9 ± 4.2 μm). For all 3 volumes of collagen used, RAFT TEs produced using 3 mg/ml collagen were thicker than those produced using 1 mg/ml collagen (*p* < 0.05). Similarly, for all 3 concentrations of collagen used, RAFT TEs produced using 2.4 ml of collagen were thicker than those produced using 0.6 ml of collagen (*p* < 0.05). Linear regression analysis revealed a positive correlation between collagen volume and RAFT TE thickness (*R*^2^ = 0.5333, *p* < 0.05).

All proximal HAM samples were thicker than distal HAM samples isolated from the same donor (n.s. *p* > 0.05). RAFT TEs produced using 0.6 ml of 1 mg/ml collagen were thinner than both proximal and distal HAM samples (*p* < 0.05), whereas RAFT TEs produced using 2.4 ml of 3 mg/ml collagen were thicker than both proximal and distal HAM samples (*p* < 0.05). All other RAFT TEs were of comparable thicknesses to HAM samples (*p* > 0.05).

### 3.3 Transparency of RAFT TEs is affected by both collagen volume and concentration, and is similar to that of HAM

**Fig. 3:**
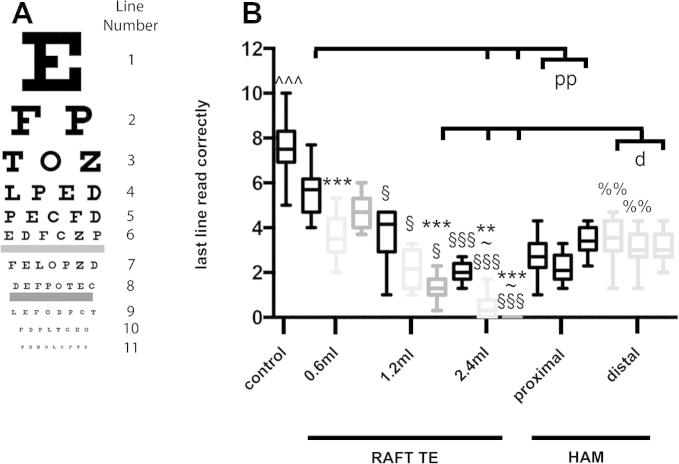
Transparency of RAFT TEs and HAM samples. A: standardized chart used in this study (left) with line numbers (right) indicated. B: RAFT TEs were produced using either 0.6 ml, 1.2 ml or 2.4 ml of 1 mg/ml collagen (black boxes), 2 mg/ml collagen (light grey boxes) or 3 mg/ml collagen (mid grey boxes). Masked observers assessed transparency of the different RAFT TEs, HAM samples (far right), and control well (far left). Data are averages from 14 masked observers: central line represents median, box represents first and third quartiles, and whiskers represent minimum and maximum. ^^^*p* < 0.005 compared to all RAFT TE and HAM samples. §*p* < 0.05, §§§*p* < 0.005 compared to RAFT TEs produced using the same concentration of collagen but 0.6 ml volume. ∼*p* < 0.05 compared to RAFT TEs produced using the same concentration of collagen but 1.2 ml volume. ^∗∗^*p* < 0.01, ^∗∗∗^*p* < 0.005 compared to RAFT TEs produced using the same volume of collagen but 1 mg/ml collagen. %%*p* < 0.01 compared to proximal HAM samples isolated from the same donor. d *p* < 0.05 distal HAM samples compared to RAFT TE. pp *p* < 0.01 proximal HAM samples compared to RAFT TE.

Transparency was greater for the control well than for any of the RAFT TE or HAM samples (*p* < 0.005) (Fig. 3). Transparency of RAFT TEs was affected by both collagen concentration and volume. For RAFT TEs produced using 1.2 and 2.4 ml of collagen, RAFT TEs produced using 1 mg/ml collagen were more transparent than those produced using 3 mg/ml collagen (*p* < 0.005). When 2.4 ml of collagen was used, this improvement in transparency was also apparent between RAFT TEs produced using 1 and 2 mg/ml collagen (*p* < 0.01). This effect was not apparent for RAFT TEs produced using 0.6 ml of collagen.

For all 3 concentrations of collagen, RAFT TEs produced using 0.6 ml of collagen were more transparent than those produced using both 1.2 ml of collagen (*p* < 0.05) and 2.4 ml of collagen (*p* < 0.005). For RAFT TEs produced using 2 and 3 mg/ml collagen, these differences were also apparent between 1.2 ml of collagen and 2.4 ml of collagen (*p* < 0.05). Linear regression analysis revealed a negative correlation between transparency and collagen volume (*R*^2^ = 0.6971, *p* < 0.01) and RAFT TE thickness (*R*^2^ = 0.5565, *p* < 0.05).

For donors 1 and 2, distal HAM was more transparent than proximal HAM (*p* < 0.01). RAFT TEs produced using 0.6 ml of 1 mg/ml collagen were more transparent than proximal HAM samples (*p* < 0.01). However, RAFT TEs produced using 2.4 ml of 2 mg/ml collagen or 3 mg/ml were less transparent than both proximal (*p* < 0.01) and distal (*p* < 0.05) HAM samples. Similarly, RAFT TEs produced using 1.2 ml of 3 mg/ml collagen were less transparent than distal HAM samples (*p* < 0.05). All other RAFT TEs were comparable to HAM samples (*p* > 0.05).

### 3.4 Light transmission through RAFT TEs is affected by both collagen concentration and volume, and transmission values approach those of HAM

**Fig. 4:**
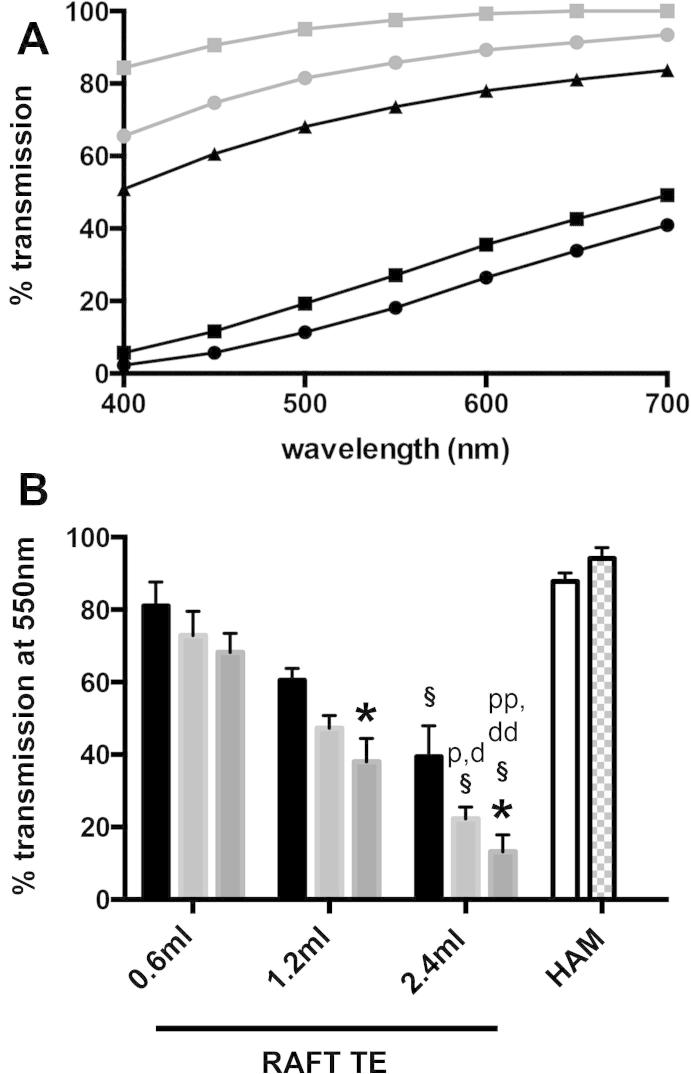
Light transmission through RAFT TEs and HAM samples. A: transmission (400–700 nm) through RAFT TEs produced using either 2.4 ml of 3 mg/ml collagen (black circles), 2.4 ml of 2 mg/ml collagen (black squares) or 0.6 ml of 3 mg/ml collagen (black triangles) was compared to transmission through proximal HAM samples (grey circles) and distal HAM samples (grey squares). For clarity, this figure shows data from a single, typical RAFT TE construct for each condition and one typical HAM donor. B: transmission (550 nm) through RAFT TEs produced using volumes of 0.6 ml, 1.2 ml or 2.4 ml of 1 mg/ml collagen (black bars), 2 mg/ml collagen (light grey bars) or 3 mg/ml collagen (mid grey bars), and proximal (white bar) and distal (checkerboard bar) HAM samples. Data are averages from 3 experimental repeats ±SD. ^∗^*p* < 0.05 compared to RAFT TEs produced using the same volume of collagen but 1 mg/ml collagen. §*p* < 0.05 compared to RAFT TEs produced using the same concentration of collagen but 0.6 ml volume. d *p* < 0.05, dd *p* < 0.01 compared to distal HAM samples. p *p* < 0.05, pp *p* < 0.01 compared to proximal HAM samples.

For both RAFT TEs and HAM samples, transmission increased with wavelength between 400 and 700 nm (Fig. 4A).

Light transmittance at 550 nm through RAFT TEs was affected by both collagen concentration and volume (Fig. 4B). RAFT TEs produced using 0.6 ml of 1 mg/ml collagen permitted most light transmission (81.1 ± 6.5%), whilst those produced using 2.4 ml of 3 mg/ml permitted the least (13.2 ± 4.7%). For RAFT TEs produced using 1.2 ml and 2.4 ml of collagen, 1 mg/ml collagen RAFT TEs transmitted more light than those produced using 3 mg/ml collagen (*p* < 0.05). For all 3 concentrations of collagen, RAFT TEs produced using 0.6 ml of collagen permitted greater transmission than those produced using 2.4 ml of collagen (*p* < 0.05). Linear regression analysis revealed a negative correlation between light transmittance and collagen volume (*R*^2^ = 0.7955, *p* < 0.005) and RAFT TE thickness (*R*^2^ = 0.7786, *p* < 0.005).

For each donor, distal HAM samples permitted greater light transmission than proximal HAM samples, but these differences were not statistically significant. Both proximal and distal HAM biopsies were more transmissive than RAFT TEs produced using 2.4 ml of 2 mg/ml collagen (*p* < 0.05) and 3 mg/ml collagen (*p* < 0.01). All other RAFT TEs were comparable to HAM samples (*p* > 0.05).

### 3.5 RAFT TE mechanical properties are affected by both collagen volume and concentration, and is similar to that of HAM

The average break force for RAFT TEs was affected by both collagen volume and concentration (Table 1). RAFT TEs produced using 0.6 ml of 2 mg/ml collagen broke under the smallest applied force (0.105 ± 0.023 N), whilst those produced using 2.4 ml of 3 mg/ml collagen broke under the greatest applied force (0.732 ± 0.098 N). Linear regression analysis revealed a positive correlation between break force and collagen volume (*R*^2^ = 0.5983, *p* < 0.05). (RAFT TEs produced using 0.6 ml of 1 mg/ml collagen were too weak to be tested accurately and so results for these are not presented.)

**Fig. 5:**
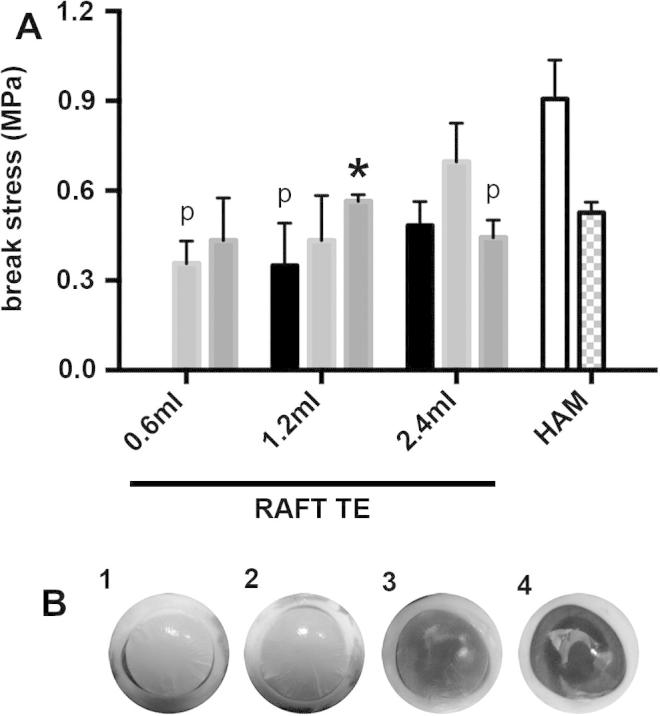
Mechanical properties of RAFT TEs and HAM samples. A: break stresses for RAFT TEs and HAM samples were calculated following testing in a custom made tensile strength testing device. RAFT TEs were produced using either 0.6 ml, 1.2 ml or 2.4 ml of 1 mg/ml collagen (black bars), 2 mg/ml collagen (light grey bars) or 3 mg/ml collagen (mid grey bars) and compared with proximal (white bar) and distal (checkerboard bar) HAM samples. (No data are presented for RAFT TEs produced using 0.6 ml of 1 mg/ml collagen as these were too weak to be tested accurately.) Data are averages from 3 experimental repeats ±SD. ^∗^*p* < 0.05 compared to RAFT TEs produced using the same volume of collagen but 1 mg/ml collagen. B: RAFT TEs were produced using either 2.4 ml of 3 mg/ml collagen (1), 2.4 ml of 2 mg/ml collagen (2) or 0.6 ml of 3 mg/ml collagen (3) and glued onto the anterior surface of an *ex vivo* porcine eye (4, without RAFT TE) using fibrin glue.

The tensile strength of RAFT TEs produced using 1.2 ml of collagen was affected by collagen concentration (Fig. 5A): RAFT TEs produced using 3 mg/ml collagen failed under greater stress than those produced using 1 mg/ml collagen (*p* < 0.05). RAFT TEs produced using 0.6 ml of 2 mg/ml collagen broke under the smallest stress (0.35 ± 0.08 MPa), whilst those produced using 2.4 ml of 2 mg/ml collagen broke under the greatest stress (0.70 ± 0.13 MPa).

For each of the 3 HAM donors, proximal HAM samples broke under a greater applied force and stress than distal HAM samples, although neither difference was statistically significant. RAFT TEs produced using 0.6 ml of 2 mg/ml collagen, 1.2 ml of 1 mg/ml collagen and 2.4 ml of 3 mg/ml collagen all failed at a lower break stress than proximal HAM samples (*p* < 0.05). All other RAFT TEs were comparable to both proximal distal HAM samples (*p* > 0.05).

### 3.6 RAFT TE with suitable optical properties for transplant can be attached to the anterior surface of an *ex vivo* porcine eye

RAFT TEs produced using either 2.4 ml of 3 mg/ml, 2.4 ml of 2 mg/ml or 0.6 ml of 3 mg/ml collagen could all withstand manipulation enabling attachment to the anterior surface of an *ex vivo* porcine eye using fibrin glue (Fig. 5B).

### 3.7 RAFT TEs and HAM biopsies degrade over time in collagenase at comparable rates

**Fig. 6:**
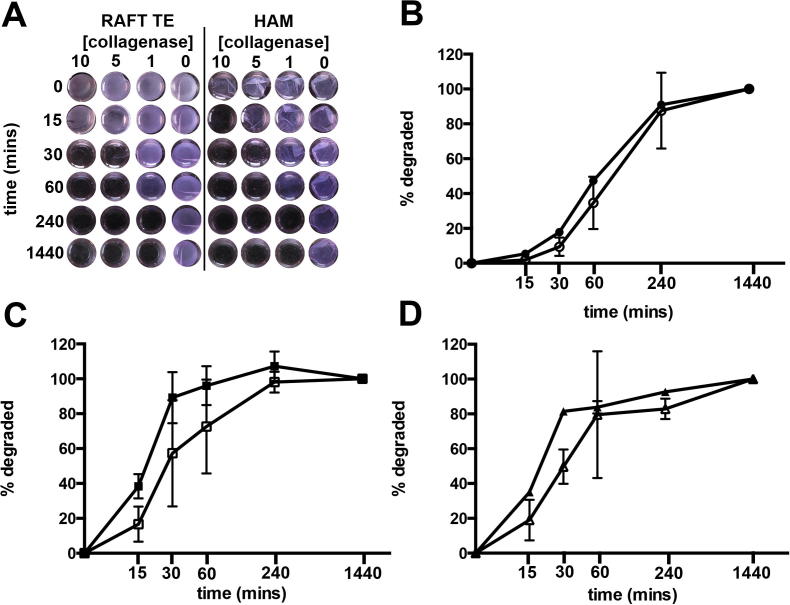
Degradation of HAM and RAFT TE in the presence of collagenase. A: representative photographs of RAFT TEs and HAM samples after 0, 15, 30, 60, 240 and 1440 min incubation with either 10 mg/ml, 5 mg/ml, 1 mg/ml or 0 mg/ml collagenase at 37 °C. B, C, and D: Quantitative measurement of degradation of RAFT TE (closed markers) and HAM samples (open markers) after incubation with 1 mg/ml collagenase (B), 5 mg/ml collagenase (C) or 10 mg/ml collagenase (D). Data are averages from 3 experimental repeats ±SEM. There were no statistically significant differences between % RAFT TE degradation and % HAM degradation at any time point using any concentration of collagenase.

Both RAFT TEs and HAM samples degraded over time following incubation with collagenase (Fig. 6). When collagenase was absent, neither RAFT TEs not HAM samples degraded (collagen concentrations in the solution remained unchanged between 0 and 1440 min, *p* > 0.05). Degradation was slowest in 1 mg/ml collagenase when for both RAFT TE and HAM samples: 50% degradation occurred after approximately 60 min. Degradation was quicker in 5 mg/ml collagenase and 10 mg/ml collagenase, where approximately 50% degradation occurred after 30 min or less. Crucially, at no time point, in any of the 3 concentrations of collagenase tested, was there a difference in degradation rate between RAFT TE and HAM samples (*p* > 0.05).

### 3.8 Human limbal epithelial cells can be expanded on the surface of optimal RAFT TEs, and transparency of RAFT TEs is maintained

**Fig. 7:**
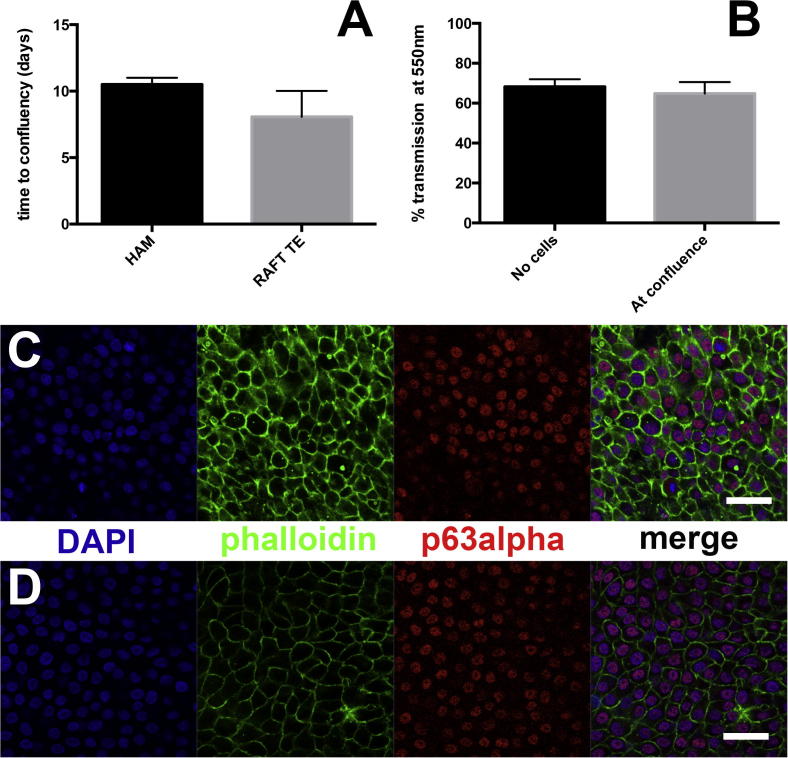
Characterization of hLE growth on HAM and RAFT TE. A: Time to confluency was measured on both HAM and RAFT TE. Data are averages of 2 donors for HAM and 6 donors for RAFT TE ±range. B: % transmission at 550 nm through RAFT TE was measured before culture (no cells) and when a confluent cell layer was present (at confluence). Data are averages of 4 donors ±SD, *p* > 0.05. C and D: wholemount immunohistochemistry was used to characterize hLE phenotype on both HAM (C) and RAFT TE (D). Images are representative of 5 fields of view captured per RAFT TE (6 donors) or HAM biopsy (2 donors). Staining shows DAPI (blue), phalloidin (green) and putative stem cell marker p63α (red). Scale bar = 50 μm.

hLE confluency was achieved on all RAFT TEs (*n* = 6 donors) but only on 2 out of 3 denuded HAM biopsies (*n* = 3 donors) (Fig. 7); results from the unsuccessful HAM cultures have been excluded from further analysis and so results are expressed as averages ± range. hLE took an average of 8.0 ± 3.0 days to reach confluency on RAFT TEs produced using 0.6 ml of 3 mg/ml collagen (*n* = 6); this is comparable to hLE expansion on denuded HAM, which took an average of 10.5 ± 0.5 days (*n* = 2). Wholemount immunohistochemistry of hLE cultures on both RAFT TEs and denuded HAM revealed that hLE were small, tightly packed, with scant cytoplasm with cobblestone morphology. Cell densities and p63α expression were also similar on each.

Importantly, transmission of light at 550 nm through RAFT TEs was maintained once cell confluency was achieved. Prior to cell seeding, transmission at 550 nm was 68.23 ± 3.68%, which did not differ from when hLE were confluent across the surface of RAFT TEs, 64.81 ± 5.70% (*p* > 0.05) (*n* = 4).

## 4 Discussion

hLE transplantation is an established approach to treating LESC deficiency (reviewed in [30,31]). However, the method of expanding and carrying hLE to the patient is yet to be optimized. One of the most commonly used carriers for LESC is HAM but clinical graft manufacture can be inconsistent – optical and mechanical properties vary, expensive screening regimes are required, and supply can be unreliable. As a result, we have developed RAFT TEs, which are already proven to support hLE expansion and stratification, suggesting that they may be clinically useful [21,23]. hLE transplantation alone should contribute towards restoration of vision but the carrier for hLE should also have appropriate optical properties so that the benefits of hLE transplantation are not counteracted. The thickness of the TE is important as, if too thin, it may be too difficult to handle and lack the required mechanical strength. Conversely, if too thick, it may project too far from the eyeball, which may be uncomfortable for the recipient and complicate the surgical procedure. In this study, we aimed to optimize the physical properties of RAFT TEs. A potential caveat to our approach is that we assume that batch-to-batch variation in our raw material, bovine hide collagen, is low. However, the processing of the collagen is tightly controlled by the suppliers’ own quality management systems, and in our own experience we have found that results are reproducible between different batches.

HAM was utilized as a comparator with RAFT TEs as it is already established in treatment of LESC deficiency. Both RAFT TEs and HAM (biopsied from locations proximal and distal to placental disc) were assessed using parameters designed to assess the feasibility of RAFT TEs for hLE transplantation. Interestingly, despite the relatively widespread use of HAM, surprisingly few studies have characterized the HAM’s physical properties and reported findings can be conflicting. Reported thicknesses of intact HAM samples vary from 60 to 200 μm [11,32] (and measurements were taken from images of from haematoxylin and eosin stained sections of formalin fixed, paraffin wax embedded samples [11], which will have undergone dehydration/rehydration cycles). Similarly, reported values for transmission of light through intact HAM samples vary between 50% and 80% at 550 nm [11,32].

In our study, we measured the thicknesses of the HAM samples and RAFT TEs using OCT, without prior fixation. It should be noted that OCT assumes that materials will have similar refractive indices. Human cornea and HAM are known to have similar refractive indices (1.33–1.37) [11,33] but the refractive index of RAFT TEs is unknown. For this reason, we confirmed our OCT measurements using pachymetry and found no significant difference in obtained thickness values (data not shown), suggesting that the refractive index of RAFT TE must be similar to cornea and HAM. In further support of this, another similar collagenous TE intended for corneal replacement has been found to have a refractive index of 1.35 [34].

In agreement with others, we found intra donor variation in HAM thicknesses [11]: proximal HAM samples tended to be thicker than distal HAM samples. Despite this trend, we found that all of the HAM samples from the 3 donors were of comparable thicknesses (∼100–150m). Similarly, we measured transmission at 550 nm to be ∼85–99% for all 3 donors, which again is more consistent, but also higher, than previously reported. However, in our study, we removed the epithelium from the HAM samples as denuded HAM (i.e. without epithelium), would be utilized for hLE expansion prior to transplant [35–38] and we wanted to compare carriers directly (i.e. denuded HAM with RAFT TE (without hLE)). This may account for the differences between our findings and those previously reported, although it is acknowledged that our sample size is relatively small.

Another important TE criterion is reproducibility. Since as many HAM samples as possible are harvested from each donor, this means that any two samples harvested from the same donor may differ from each other (confirmed when we compared samples taken from locations proximal and distal to placental disc, differences were most evident for mechanical properties). Along with the expected inter donor variation, this intra donor variation may further complicate hLE expansion protocols and surgical procedures, and may affect clinical outcome. In contrast, the process of RAFT TE production is simpler as expensive screening regimes are not required, collagen supply is more reliable, and a standardized product with known physical properties can be achieved. This is useful in terms of Good Manufacturing Practice protocol development and also important as it simplifies development of quality controls, potency assays [39] and decisions regarding release criteria.

The RAFT TE production process is also tunable. By varying the concentration and volume of collagen utilized to produce hydrogels, RAFT TEs with differing optical and mechanical properties were achieved. In general, collagen concentration and volume increased RAFT TE thickness and tensile strength, and decreased light transmission and transparency. Overall, the total amount of collagen impacted upon RAFT TE optical properties (i.e. RAFT TEs produced using 2.4 ml of 1 mg/ml collagen or 1.2 ml of 2 mg/ml collagen (both a total of 2.4 mg collagen) were comparable). Encouragingly, the optical properties of the vast majority of RAFT TEs produced using different amounts of collagen did not differ significantly from HAM samples, except when the very largest or smallest amounts of collagen were used.

When we looked at the mechanical properties of RAFT TEs, we found that the RAFT TEs produced using 2 smallest amounts of collagen (excluding those produced using 0.6 ml of 1 mg/ml collagen, which could not be handled) were significantly weaker than proximal HAM samples, but were still comparable to distal HAM samples. Interestingly, RAFT TEs produced using 2.4 ml of 3 mg/ml collagen withstood the greatest applied force of all RAFT TEs before failure, but this equated to a lower break stress than anticipated, and was instead similar to RAFT TEs produced using 1.2 ml of 2 mg/ml collagen, a total of 3-fold less collagen. This may reflect that dehydration of this hydrogel containing such a large amount of collagen was incomplete after application of the hydrophilic porous absorber. This hypothesis is supported by the fact that this RAFT TE was far thicker than any of the other RAFT TEs tested in this study.

This capability to produce RAFT TEs with different properties is useful and highlights that RAFT TEs may be useful in other regenerative medicine applications where different properties may be prioritized. Here for ocular surface, we aimed to produce a thin, transparent TE but where a stronger TE is required, for artificial skin perhaps, a thicker, stronger, less transparent TE may be more appropriate. In this study, we identified that RAFT TEs produced using 0.6 ml of 3 mg/ml collagen provided the best optical properties but were still strong enough to be attached to an eye, indicating that this protocol provided the optimal RAFT TE for treatment of LESC deficiency.

In order to confirm the potential clinical applicability of these ‘optimal’ RAFT TEs, it was necessary to investigate their biodegradation over time. HAM is known to degrade following transplantation [40]; this is useful since this can further improve visual acuity, as the path of light through the cornea is not interrupted by the interface between HAM and native stroma. Whilst it is difficult to reproduce the *in vivo* environment *in vitro*, matrix-metalloproteinases (including collagenases) are present in corneas – indeed at elevated levels in diseased or inflamed corneas [41] – which likely contribute to the biodegradation of HAM over time. Importantly, we found that the ‘optimal’ RAFT TEs and HAM biopsies degraded at comparable rates *in vitro* across a range of collagenase concentrations. Additionally, the rate of RAFT TE degradation was much more uniform than for HAM, where samples from different donors degraded at differing rates when exposed to the same concentration of collagenase. That biodegradation rates of RAFT TE are comparable with HAM is useful as clinicians will be familiar with this. Additionally, that the rate of RAFT TE degradation is more uniform may provide further clinicians with further confidence during follow-up after hLE transplantation.

We also sought to compare hLE cultures on ‘optimal’ RAFT TEs with hLE cultures on HAM. We found that hLE expansion times on either substrate did not differ, which is important given that culture is expensive, time-consuming and delays patient treatment. We also found that transparency of the RAFT TEs was unaffected by the presence of a confluent hLE layer, which is important as this would negate producing a more transparent RAFT TE. Crucially, we found that hLE morphology and p63α expression was similar on RAFT TEs and HAM: hLE on both were tightly packed and displayed a cobblestone morphology, with scant cytoplasm and high levels of putative stem cell marker p63α. These characteristics are typical of undifferentiated cells: limbal epithelial stem cells or their progeny, transient amplifying cells, both of which contribute to corneal regeneration *in vivo*. On RAFT TEs, expression of corneal epithelial differentiation markers, such as cytokeratins 3/12, is typically only observed in superficial cell layers following culture at the air/liquid interface which triggers spontaneous stratification [23]. In this case, given that the expression of p63α was so widespread, it seems likely that the majority of the cells were in fact transient amplifying cells as opposed to limbal epithelial stem cells. Since high levels of p63α expression (i.e. a high number of undifferentiated cells) have previously been demonstrated to be important in *in vitro* potency assays on RAFT TEs [39,42], these data further support that this optimal RAFT TE would be a suitable substrate for hLE transplantation in the treatment of LESC deficiency.

One potential advantage of HAM over RAFT TE, that is not considered in this study, is that HAM is often reported to release soluble factors that promote hLE proliferation and reduce neovascularization post transplantation [43–46]. However, to treat LESC deficiency effectively, hLE must be transplanted which requires *in vitro* pre expansion either on HAM or RAFT TEs (this therapeutic approach is more complex than alternative protocols where HAM is simply used to patch epithelial defects and hLE transplantation is unnecessary [47]). It is possible that during hLE pre expansion the beneficial soluble factors from HAM are diluted out or even “swamped” by those present in serum of culture medium. Additionally, supporting cells can be incorporated into RAFT TEs and it has been demonstrated that these cells enhance hLE phenotype on RAFT TEs *in vitro* [23], presumably via release of soluble factors. Furthermore, once transplanted, hLE will be in close proximity to the supporting cells of, and secreted factors from, the recipient’s eye, which, again, may improve clinical outcome.

## 5 Conclusions

LESC deficiency can cause blindness. Whilst hLE transplantation is an established treatment for this disease, the method of delivery is yet to be optimized. We have now demonstrated that the RAFT TE production process is tunable, which is advantageous here as optical and mechanical properties suitable for use on the front of the eye can be made. RAFT TEs produced using 0.6 ml of 3 mg/ml collagen were thin, and transparent but strong enough to withstand attachment to an eye. Moreover, these RAFT TEs degraded at similar rates to HAM and were capable of supporting hLE cultures *in vitro*, suggesting that this may be useful towards treatment of LESC deficiency. However, the RAFT TE production process is also tunable and may be easily altered in other regenerative medicine applications where other properties may be prioritized.

## Disclosures

IM, AKK, SS, AJS none; JTD holds peer reviewed funding for development of the RAFT process.

## Figures and Tables

**Table 1:** Tensile strength testing of RAFT TEs and HAM samples.

	Collagen concentration (mg/ml)	Collagen volume (ml)	Average break force (Newtons) ± standard deviation
RAFT TE	1	0.6	–
	2		0.105 ± 0.023
	3		0.167 ± 0.055
		
	1	1.2	0.121 ± 0.049
	2		0.216 ± 0.077
	3		0.353 ± 0.014
		
	1	2.4	0.261 ± 0.044
	2		0.585 ± 0.109
	3		0.732 ± 0.098

	HAM donor number	Sample location	

HAM	1	Proximal	0.544 ± 0.046
		Distal	0.225 ± 0.055
		
	2	Proximal	0.490 ± 0.057
		Distal	0.245 ± 0.085
		
	3	Proximal	0.408 ± 0.075
		Distal	0.196 ± 0.049

## Appendix A Figures with essential colour discrimination

Certain figures in this article, particularly Figs. 6 and 7, are difficult to interpret in black and white. The full colour images can be found in the on-line version, at doi: http://dx.doi.org/10.1016/j.actbio.2015.06.007.
